# Dynamics of heart rate variability in patients with type 2 diabetes mellitus during spinal anaesthesia: prospective observational study

**DOI:** 10.1186/s12871-015-0125-6

**Published:** 2015-10-08

**Authors:** Su Hyun Lee, Dong Hoon Lee, Dong Hoon Ha, Young Jun Oh

**Affiliations:** Department of Anaesthesiology and Pain Medicine, Yonsei University College of Medicine, 50 Yonsei-ro, Seodaemun-gu, Seoul, 120-752 Korea

**Keywords:** Spinal anaesthesia, Heart rate variability, diabetes mellitus

## Abstract

**Background:**

Little is known about the changes in autonomic function during spinal anaesthesia in type 2 diabetic patients. The purpose of the study was to assess the influence of spinal anaesthesia on the heart rate variability in type 2 diabetic patients according to the glycated hemoglobin (HbA_1c_) level.

**Methods:**

Sixty-six patients who were scheduled for elective orthostatic lower limb surgery were assigned to three groups (n = 22, each) according to HbA_1c_; controlled diabetes mellitus (HbA_1c_ < 7 %), uncontrolled diabetes mellitus (HbA_1c_ > 7 %) and the control group. The heart rate variability was measured 10 min before (T0), and at10 min (T1), 20 min (T2) and 30 min (T3) after spinal anaesthesia.

**Results:**

Before spinal anaesthesia, total, low-and high-frequency power were significantly lower in the uncontrolled diabetec group than in other group (p < 0.05). During spinal anaesthesia, total, low- and high-frequency powers were did not change in the uncontrolled diabetec group while the low-frequency power in the controlled diabetec group was significantly depressed (p < 0.05). The ratio of low-to high-frequency was comparable among the groups, while it was reduced at T1-2 than at T0 in all the groups. The blood pressures were higher in the uncontrolled diabetec group than in the other groups.

**Conclusions:**

Spinal anaesthesia had an influence on the cardiac autonomic modulation in controlled diabetec patients, but not in uncontrolled diabetec patients. There were no differences in all haemodynamic variables during an adequate level of spinal anaesthesia in controlled and uncontrolled type 2 DM.

**Trial registration:**

ClinicalTrials.gov NCT02137057

**Electronic supplementary material:**

The online version of this article (doi:10.1186/s12871-015-0125-6) contains supplementary material, which is available to authorized users.

## Background

Type 2 diabetes mellitus (DM) is a widespread disease and increases the risk of cardiovascular disease [1], peri-operative hypotension [2, 3] and intra-operative morbidity [4]. Hyperglycemia in type 2 diabetes is associated with micro- and macro-complications [5] and causes autonomic nervous dysfunction. Severe autonomic failure due to sympathetic and parasympathetic dysfunction typically occurs in patients with long-standing and poorly controlled diabetes [6]. The incidence of caused by uncompensated vasoconstriction during general anaesthesia was highly reported in patients with autonomic nervous dysfunction due to diabetes [2]. Therefore, these patients present a major challenge to the anaesthetist. Strict glycemic control can decrease the morbidity rate for various complications of type 2 diabetes, thus the glycated hemoglobin (HbA_1c_) level is recommended below 7 % [7].

Heart rate variability (HRV) is a standard screening parameter for diagnosis of autonomic dysfunction [8, 9]. The total power reflects the overall autonomic modulation [10, 11] and is considered an estimation of the balance between sympathetic and parasympathetic nerve activities. Several studies have reported linear or non-linear correlations between chronic hyperglycemia and HRV [12–15].

The blockade of pre-ganglionic sympathetic fibres and cardiac sympathetic innervation during spinal anaesthesia can induce central sympatholysis. Therefore, the effects of sympathetic blockade on cardiac autonomic nervous function in type 2 diabetes are expected to be different from that in normal patients.

Previous studies have reported that hypotension during spinal anaesthesia can be predicted by changes in the ratio of low to high frequency power [16, 17], although the subjects in their studies were not DM patients and the blood pressure (BP) changes according to HbA_1c_ levels were not verified during spinal anaesthesia.

Little is known about changes in cardiac autonomic nervous function during spinal anaesthesia in type 2 diabetes. In addition, there was not enough evidence whether bradycardia and hypotension occur more frequently with uncontrolled diabetes patients than controlled diabetes or normal patients during spinal anaesthesia. In type 2 diabetes patients, although the specific mechanisms have not been established, it is clear that sympathetic blockage caused by spinal anaesthesia has effects on the cardiac autonomic system. The primary outcome of our study was to evaluate the change of cardiac autonomic system in spinal anaesthesia using the component of HRV in type 2 diabetes patients comparing with non-diabetic patients.

## Methods

This study received approval from the institutional review board of Severance Hospital, Yonsei University Health System, Seoul, South Korea (Ref. 4-2014-0167) on April 29, 2014 and was registered at ClinicalTrials.gov (NCT 02137057). This prospective observational study followed the STROBE guideline (Additional file 1). A total of 66 adult patients aged 40–80 years with an American Society of Anaesthesiologists physical status of I–III who were scheduled to undergo elective lower limb orthostatic surgery were enrolled after obtaining informed written consent. The exclusion criteria comprised contraindication to spinal anaesthesia, patients with arrhythmia and those taking medications that affect the autonomic nervous system, such as anti-depressants, hypnotics, β- and α-adrenergic blockers.

### Patient’s allocation

Patients were allocated into their respective groups by setting HbA_1c_ 7 % as our parameter for patients previously diagnosed with DM in the following matter: controlled DM (HbA_1c_ < 7 %), uncontrolled DM (HbA1c > 7 %) and normal groups (n = 22, each). Normal patients were defined as never having a previous diagnosis of DM and a fasting randomized sugar level < 200 and thereby not fitting the criteria for impaired glucose tolerance.

### Anaesthetic management

No patient was pre-medicated. All patients were monitored using electrocardiography, non-invasive BP measurements and pulse oximetry from arrival in the operating room. For HRV analysis, PowerLab (AD Instruments, Sydney, NSW, Australia) was used to automatically save cardiac electrophysiology measurements. The day before surgery, it is standard to start fasting at midnight and dehydration that comes with this is supplemented with NS 5 ml/kg before spinal anaesthesia. This is the standard care at our hospital.

All patients were stabilized for 20 min. For spinal anaesthesia, a 22 G spinal needle aimed at the lumber (L)3–4 or L4-5 inter-vertebral space was used, and with the patients in a lateral position, 10–12 mg of 0.5 % hyperbaric bupivacaine was injected into the subarachnoid space. After spinal injection, a infusion of 6 % hydroxyethyl starch (Voluven®; Fresenius Kabi, Bad Homburg, Germany) was started at a rate of 5 ml/kg.

The level of spinal block was confirmed by the loss of pain sensation after a pin-prick test, with the target level being T6–8. Baseline blood pressure and pulse rate were recorded in the supine position 10 min before and at 10, 20 and 30 min after spinal anaesthesia. Hypotension was defined as a systolic blood pressure < 90 mmHg, and was managed by 4 mg of ephedrine gradually injected until normalization of systolic blood pressure. Bradycardia was defined as a heart rate (HR) of < 50 beats.min^−1^ based on a consecutive recording of ≥15 sec, and was managed by administering 0.25–0.5 mg of atropine.

### HRV analysis

HRV analysis was conducted using LabChart v7 software (AD Instruments, Sydney, NSW, Australia) according to the Task Force of the European Society of Cardiology and the North American Society of Pacing and Electrophysiology recommendations [10]. During the recording, using a sampling rate of 1000H as a threshold method, we evaluated the fast peak R wave of ECG. A computer was used to record and analysis the beat to beat variability of consecutive R wave of sinus rhythm. Abnormal RR interval of premature beat of artifact that included noise was automatically removed by LabChart. The low-frequency (LF, 0.04–0.15Hz) component of HRV is mediated by both sympathetic and parasympathetic nerve activities, while the high-frequency (HF, 0.15–0.4 Hz) component is mediated almost entirely by parasympathetic nerve activity. The total power (TP, 0–0.4 Hz) reflects the overall autonomic activity [10, 11] and LF/HF ratio verifies the balance between sympathetic and parasympathetic nerve activities. Frequency-domain analysis of HRV was conducted using data from 5 min segments without ectopic beats and artefacts using fast-Fourier transformation. Time points were set at 10 min before (T0), 10 (T1), 20 (T2) and 30 min (T3) after spinal anaesthesia.

### Statistical analysis

Data from a previous study gave a mean (SD) of 630 (190) ms^2^ of TP in DM patients [18]. We estimated that 20 patients in each group would be required to detect a mean difference of 120 ms^2^ and standard deviation 190 ms^2^ in the TP between DM groups and normal group with a power of 80 % at a significance of *P* < 0.05. To compensate for possible dropouts, we included 22 patients per group. All variable data were tested for normality using the Kolmogorov-Smirnov test. For inter-groups comparisons, one-way ANOVA with Bonferroni correction, chi-square or Fisher’s exact test were used. For intra-group comparisons of HRV and haemodynamic parameters compared to each baseline values, repeated measures analysis of variance with Bonferroni corrections was used. Statistical analyses were performed using SPSS 20.0 software (SPSS Inc., Chicago, IL, USA) and a p value of < 0.05 was considered statistically significant.

## Results

**Fig. 1:**
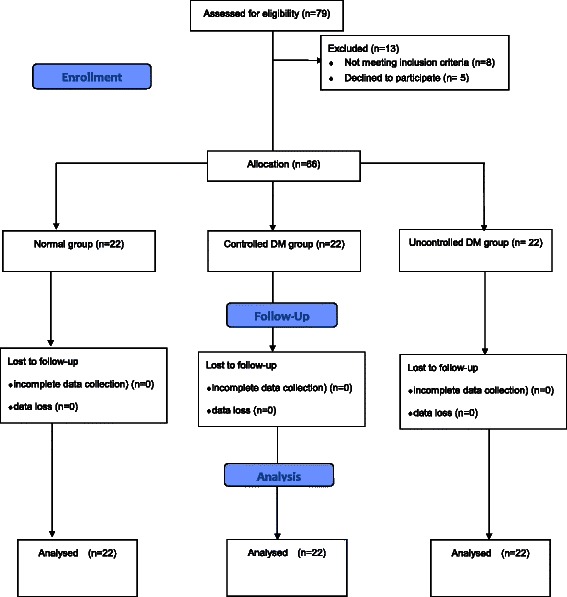
Study process of showing this trial

Seventy nine patients were determined to be eligible for this study, but 5 of them were excluded due to decline to participate this study and 8 were not studied due to history of arrhythmia or those taking medications that affect the autonomic nervous system (Figure 1). There were no differences of the patients’ age, body mass index and sex (Table 1). The median peak block level was T 6 ± 2 with no difference among the three groups. The HbA1c level was 8.5 ± 1.0 % in uncontrolled DM and 6.5 ± 0.4 % in controlled DM. The incidence of hypotension and bradycardia was not different among the groups. In addition, the given number of ephedrine was not differentiated between the groups (Table 1).

**Table 1:** Characteristics of patients

	Normal group (*n* = 22)	Controlled DM (*n* = 22)	Uncontrolled DM (*n* = 22)
Age (years)	58.7 ± 9.7	64.9 ± 7.5	62.2 ± 10.3
Male/female	9/13	10/12	8/14
Height (cm)	160.8 ± 8.1	161.1 ± 8.5	163.0 ± 10.8
Weight (kg)	65.1 ± 9.5	66.4 ± 10.6	66.1 ± 13.8
BMI (kg/m^2^)	25.2 ± 4.1	25.4 ± 10.6	24.7 ± 4.0
DM duration (years)	NA	7.3 ± 5.8*	15.8 ± 8.1*
HbA_1c_ (%)	5.2 ± 0.4	6.5 ± 0.4*	8.5 ± 1.0*
ASA classification 1/2/3	12/8/2	9/10/3	0/14/8
DM medication			
Oral hypoglycemics	NA	11	7
Insulin	NA	7	7
Combined (oral + insulin)	NA	4	8
Blocked sensory T level	6 ± 2	6 ± 2	6 ± 2
Type of operation (*n*)			
Amputation (toe or knee)	0	2	7
Open reduction of fracture	10	3	5
Arthroscopic surgery	3	5	0
Debridement and curettage	2	4	5
Removal of fixation device	7	5	1
Bipolar hemiarthroplasty	0	3	4
Ephedrine given (*n*)	2	2	2
Hypertension (*n*)	2 (9 %)	4 (18 %)	2 (9 %)
CAN	NA	1 (4 %)	4 (18 %)*

Values are expressed as mean ± SD or number (proportion). DM, type 2 diabetes mellitus; BMI, body mass index; HbA_1c,_ glycated hemoglobin; T, thoracic level; CAN, cardiac autonomic neuropathy

^*^P < 0.05 compared to normal group

### Changes in heart rate variability

**Fig. 2:**
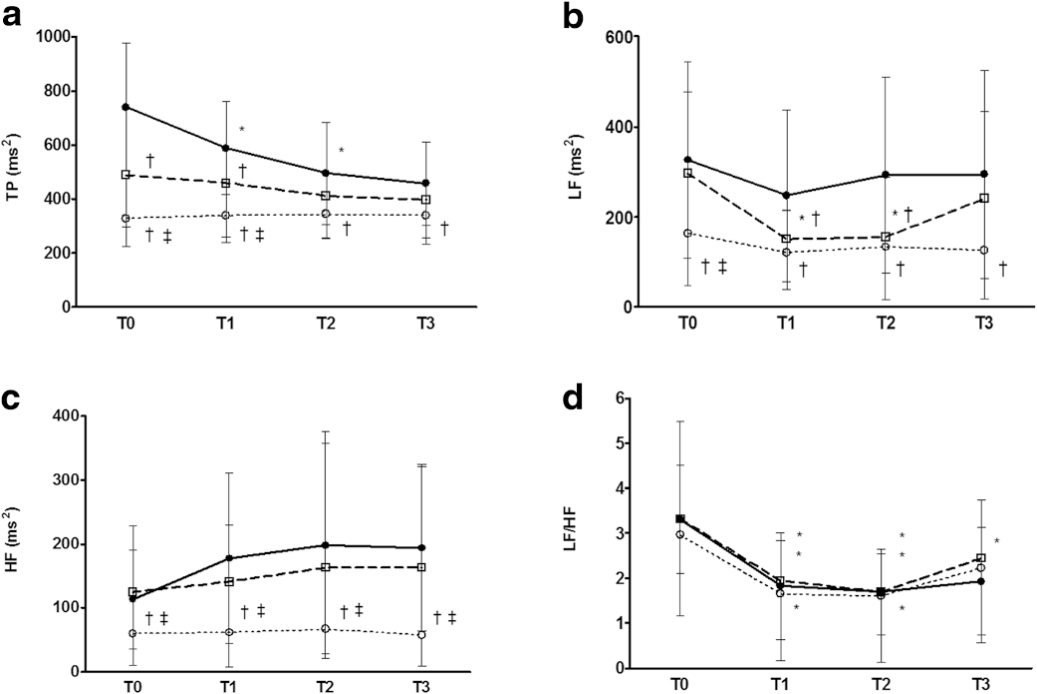
Heart rate variability measurement (**a**) total power (**b**) low-frequency (LF) power (**c**) high-frequency (HF) power (**d**) LF/HF ratio. Data are mean with error bars showing SD. T0, 10 min before spinal anaesthesia; T1, 10 min; T2, 20 min; T3, 30 min after spinal anaeshthesia; normal group N (●),controlled DM group (□), uncontrolled DM group (○). ^*^P < 0.05 compared to T0; ^†^*P* < 0.05 compared to normal group; ^‡^*P* < 0.05 compared to controlled DM group

The total, LF and HF powers were lower in the uncontrolled DM than in the other groups at T0 (*p* < 0.05). The total power of controlled DM was significantly lower than that of the normal group and significantly higher than that of the uncontrolled group at T0-1. The LF power in the controlled DM group decreased significantly and was lower than that of the normal group at T1–2. The LF power was not changed but significantly lower in uncontrolled DM group than in the normal group at T1–3. The HF power was significantly higher than that in the uncontrolled DM at T1-T3. There were no differences in the LF/HF ratio among the three groups, with the LF/HF ratio reduced at T1-2 than at T0 in all groups (Table 2; Fig. 2).

**Table 2:** Changes of heart rate variability

	Group	T 0	T1	T2	T3
Total power (ms^2^)	Normal group	740.0 ± 235.5	587.3 ± 171.7^*^	495.1 ± 189.7*	511.2 ± 123.9^*^
	Controlled DM	487.5 ± 188.4^†^	458.8 ± 200.2^†^	410.9 ± 157.9	396.1 ± 163.4
	Uncontrolled DM	328.9 ± 105.0^†,‡^	338.5 ± 100.3^†,‡^	344.0 ± 88.5^†^	339.6 ± 84.4^†^
LF power (ms^2^)	Normal group	326.4 ± 219.0	247.2 ± 190.5	292.6 ± 217.2	294.2 ± 230.0
	Controlled DM	296.2 ± 182.4	150.8 ± 64.5^*,†^	155.6 ± 131.0^*,†^	240.2 ± 194.2
	Uncontrolled DM	163.8 ± 115.3^†,‡^	121.4 ± 83.8^†^	133.8 ± 117.3^†^	126.5 ± 108.0^†^
HF power (ms^2^)	Normal group	113.2 ± 77.5	177.5 ± 133.1	198.0 ± 177.5	193.9 ± 130.2
	Controlled DM	124.5 ± 104.3	140.9 ± 88.4	162.7 ± 194.3	163.4 ± 157.6
	Uncontrolled DM	59.8 ± 49.8^†,‡^	61.5 ± 54.0^†,‡^	66.6 ± 39.3^†,‡^	57.5 ± 48.1^†,‡^
LF/HF ratio	Normal group	3.3 ± 1.2	1.8 ± 1.2^*^	1.7 ± 0.9^*^	2.1 ± 1.8
	Controlled DM	3.0 ± 1.8	1.7 ± 1.5^*^	1.6 ± 1.5^*^	2.2 ± 1.7
	Uncontrolled DM	3.3 ± 2.2	1.9 ± 0.9^*^	1.7 ± 0.8^*^	2.4 ± 1.3

Values are expressed as mean ± SD. DM, type 2 diabetes mellitus; LF, low-frequency power; HF, high-frequency power; T0, 10 min before spinal anaesthesia; T1, 10 min; T2, 20 min; T3, 30 min after spinal anaeshthesia

*P < 0.05 compared to T0

^†^*P* < 0.05 compared to normal group

^‡^*P* < 0.05 compared to controlled DM group

### Changes in haemodynamic

The systolic, mean and diastolic BP showed no differences among the three groups at T0. The systolic, mean and diastolic BP at T1-3 were reduced than that at T0 in all groups. The systolic, mean and diastolic BP of uncontrolled DM group at T1-3 were higher than in the other groups. However there were no differences in HR at T0-3 among the three groups. The HR was reduced at T3 than at T0 in the normal group and reduced at T2 and T3 than at T0 in controlled and uncontrolled DM group. The pulse pressure for uncontrolled DM was highest among the groups, but was not statistically different at T0; it remained significantly higher in uncontrolled DM compared with the other groups at T1–3 (Table 3). There were no differences in all haemodynamic variables between the controlled DM and normal groups.

**Table 3:** Changes in haemodynamics

	Group	T 0	T1	T2	T3
Heart rate	Normal group	70.5 ± 11.9	70.5 ± 13.7	65.5 ± 15.8	62.3 ± 14.6^*^
(Beats per minute)	Controlled DM	73.7 ± 12.8	73.5 ± 16.0	67.4 ± 16.1^*^	65.9 ± 15.4^*^
	Uncontrolled DM	77.5 ± 12.3	74.7 ± 13.6	72.8 ± 12.8^*^	71.6 ± 13.0^*^
Systolic blood pressure	Normal group	144.1 ± 16.3	122.4 ± 14.0^*^	118.9 ± 14.2^*^	119.0 ± 14.4^*^
(mmHg)	Controlled DM	144.5 ± 20.2	119.2 ± 19.3^*^	118.6 ± 18.6^*^	118.3 ± 19.9^*^
	Uncontrolled DM	152.9 ± 19.4	134.6 ± 22.1^*,†.‡^	134.6 ± 25.0^*,†.‡^	137.2 ± 20.2^*,†.‡^
Mean blood pressure	Normal group	111.1 ± 13.0	94.9 ± 12.5^*^	92.1 ± 10.8^*^	91.8 ± 11.3^*^
(mmHg)	Controlled DM	110.5 ± 15.1	89.8 ± 14.1^*^	91.6 ± 14.3^*^	90.9 ± 14.7^*^
	Uncontrolled DM	113.5 ± 14.3	103.6 ± 17.6^*,‡^	102.7 ± 18.1^*,‡^	105.1 ± 16.5^*,‡^
Diastolic blood pressure	Normal group	83.5 ± 11.0	73.5 ± 9.3^*^	71.2 ± 8.9^*^	71.2 ± 9.8^*^
(mmHg)	Controlled DM	84.4 ± 9.1	71.0 ± 10.9^*^	72.3 ± 12.4^*^	70.9 ± 11.7^*^
	Uncontrolled DM	84.4 ± 12.0	78.9 ± 15.4^*,†.‡^	78.6 ± 14.2^*,†.‡^	79.4 ± 14.2^*,†.‡^
Pulse pressure	Normal group	59.9 ± 11.9	47.7 ± 8.1	47.1 ± 11.8	46.9 ± 11.1
(mmHg)	Controlled DM	60.1 ± 17.1	48.1 ± 11.6	46.3 ± 11.3	47.4 ± 12.1
	Uncontrolled DM	68.5 ± 16.4	58.7 ± 16.4^†,‡^	59.0 ± 16.4^†,‡^	57.8 ± 16.1^†,‡^

Values are expressed as mean ± SD. DM, type 2 diabetes mellitus; LF, low-frequency power; HF, high-frequency power; T0, 10 min before spinal anaesthesia; T1, 10 min; T2, 20 min; T3, 30 min after spinal anaeshthesia

*P < 0.05 compared to T0

^†^*P* < 0.05 compared to normal group

^‡^*P* < 0.05 compared to controlled DM group

## Discussion

Our study provides a description of autonomic nervous system modulation on cardiac activity among diabetic patients undergoing spinal anaesthesia. The original findings of this study are that patients with uncontrolled DM have a lower baseline total, LF and HF power than the controlled DM and normal groups. We expected that in DM patients, who have affected autonomic nerve systems, spinal anaesthesia would have a greater effect on the autonomic function. However, as seen in our results, after spinal anaesthesia, the LF/HF ratio, which represents the balance of the autonomic system, was well maintained. Compared to the uncontrolled DM group, the controlled DM group had more a similar LF/HF ratio change to the normal patient group. In addition, the uncontrolled DM group, which have a decreased power of HRV component, as the LF/HF ratio was recovered, the autonomic nerve modulation was preserved. Haemodynamic stability was well maintained during spinal anaesthesia in controlled and uncontrolled DM groups.

The total power reflects the overall autonomic activity [10, 19]. Singh et al. stated the more severe the hyperglycemia, the more the total power decreases [13]. The correlations of the degree of hyperglycaemia with total, LF and HF power have been reported [12–15, 20]. In our study, the total, we were depressed in type 2 DM with uncontrolled hyperglycemia. Chronic hyperglycemia affects vascular dynamics along with oxidative stress and plays a key role in the pathogenesis of autonomic cardiac neuropathy [21, 22] and arterial stiffness [23, 24]. In this study, differences of total, LF and HF power during spinal anaesthesia were definite between controlled and uncontrolled DM, which were based on a standard HbA_1c_ level of 7 %. We found an association between high HbA1c level and low power of HRV component during spinal anaesthesia. Consistent with the findings of previous studies [2, 25], the LF/HF ratio showed no significant differences among the three groups. Similar to our results, Istenes et al. could not find statistical significance between LF/HF ratio with type 2 DM [25]. We performed thoracic spinal anaesthesia (median sensory block; range, T6–8) in this study, although a sympathetic block can extend above the 2–6 dermatome compared with sensory loss [26]. Therefore, the sympathetic block can be at 2–6 dermatomes higher than the checked blocked sensory level. Also, as the autonomic nervous system is maintained by a balance of the sympathetic and parasympathetic system, the increase of HF can not be soley credited to rise of vagal tone as we must consider the suppression or inhibition of the sympathetic modulation as a factor. A decrease in the LF/HF ratio and BP at 10 min after spinal anaesthesia seemed to occur in all three groups because the cardiac sympathetic tone of the myocardium was weakly blocked during spinal anaesthesia. We concluded that the autonomic nervous modulation was already markedly decreased and that spinal anaesthesia itself did not affect autonomic nervous function in the uncontrolled DM group, thus the cardiac autonomic modulation, which typically associated with spinal anaesthesia, did not occur. This suggests that cardiac autonomic nervous regulation is reduced in patients with uncontrolled DM during spinal anaesthesia without any significant change in the sympatho-vagal balance. In contrast, we confirmed that spinal anaesthesia significantly affected the autonomic nervous modulation in the controlled DM and normal groups. The systolic and mean BP after spinal anaesthesia was significantly higher in uncontrolled DM than in the other groups, indicating that sympatho-vagal function disturbance is not exacerbated under the adequate spinal block in the uncontrolled DM and the incidence of was comparable with controlled DM and normal group. We also observed an elevated pulse pressure in the patients with uncontrolled DM, which may reflect arterial stiffness. Various studies reported that a high systolic blood pressure in DM can be attributed to the effects of hyperglycaemia on the vascular endothelium and peripheral nerves through its interaction with vascular and metabolic factors [21, 22]. Arterial stiffness and thickness caused by vascular dysfunction in type 2 DM are correlated with an increase in systolic BP [23, 27]. Furthermore, four patients in uncontrolled DM were diagnosed with cardiac autonomic neuropathy [28] in our study. Therefore, the high systolic BP and pulse pressure both reflect arterial stiffness secondary to neuropathy and associated vascular damage.

DM patients with cardiac autonomic neuropathy who received general anaesthesia experienced a more profound decrease in HR and BP and an increased use of vasoactive drugs during induction [2]. This probably occurred due to the lack of compensatory mechanisms for the vasodilatory effect against systemically administered anaesthetics. Cardiac autonomic neuropathy is induced by several processes, including metabolic insult to nerve fibres, neurovascular insufficiency and the formation of advanced glycated end products [28]. Cardiac autonomic neuropathy involves the sympathetic and parasympathetic nervous systems depending on disease progression. As a result, HR remains constant, the coronary vasodilatory reserve is impaired, the micro-circulation decreases, and diastolic dysfunction occurs [29]. Therefore, the administration of an adequate level of spinal anaesthesia instead of general anaesthesia to patients with diabetic cardiomyopathy who exhibit normal systolic function and impaired diastolic function [30] might lead to and the ratio of low to high frequency a lesser impact on sympatho-vagal balance.

In addition to the above factors, this study has several limitations. First, patients were divided according to HbA_1c_ levels, not to the presence of cardiac autonomic neuropathy. The prevalence of cardiac autonomic neuropathy in type 2 DM patients is 34 % [28], and it becomes more evident 18 months after DM diagnosis [31]. We focused the changes in autonomic nervous function targeting HbA_1c_, because diagnosis for cardiac autonomic neuropathy was not performed in all DM patients and HbA_1c_ is used as a standard for treatment [7]. Second, patients who were taking β-blockers, angiotensin receptor blockers or angiotensin-converting enzyme inhibitors [32, 33], which can affect the autonomic nervous system, were excluded from this study; however, HRV depression by hypertension itself, including the LF and HF components, may have influenced our results [34]. Furthermore, DM patients are subjected to concurrent multiple drug use, therefore the combined effect of various drugs on the autonomic nervous system could inflict on our result. Third, systemic vascular resistance was not verified and vascular stiffness was not measured. Because DM patients with cardiac autonomic neuropathy exhibit central and peripheral vascular dysfunction [32], a decrease in systemic vascular resistance due to sympathetic block by spinal anaesthesia could be more severe [35]. Fourth, we analyzed sequences of 5 min. Our study examined the changes of HRV for a short duration of time of 30 minutes before and after spinal anaesthesia. Since the heart rate (R-to-R intervals) was different among studied patients, differences in length of tachograms can influence the derived power spectra patterns. Thereby, short sequences can underestimate LF component and overestimate the rapid fluctuations (HF component).

There are several confounding factors regarding HRV in our study. The HF power decreases in elderly patients, and the LF/HF ratio shows a positive correlation with age [36, 37]. Moreover, females show a decreased LF/HF ratio compared with males [20, 37]. Because our study aimed to verify changes in autonomic function during SA in DM patients, HRV could only be analysed during surgery. Surgery-related anxiety or emotional stress may also affect HRV [38]. Because HRV is affected by PaCO_2,_ the breathing pattern may have influenced our results [39]. The central frequency of the HF band of the heart rate coincides with the central frequency of the respiratory rate. If the respiratory rate is low (for example 10 breaths per min, corresponding to a frequency of 0.17 Hz, very near to the upper limit of LF), some amount of HF band overlaps with the LF band, thus the exact estimation of both bands becomes difficult. We need to consider the fact that DM patients with CAN lack HRV during deep breathing [29]. The HF power is mainly driven by respiration, and this causes vagal-mediated respiratory sinus arrhythmia [33]. The magnitude of the HF power is largely influenced by the depth of respiration, and this must be considered during SA.

## Conclusions

Our findings suggest that spinal anaesthesia had an influence on the cardiac autonomic modulation in controlled DM patients. Cardiac autonomic modulation did not change during spinal anaesthesia for lower limb surgery in uncontrolled DM patients who had a lower baseline autonomic nervous system modulation. However, there was no severe caused by a disturbance of sympatho-vagal function during the adequate spinal anaesthesia in controlled and uncontrolled type 2 DM.

## Additional file

Additional file 1: **STROBE guideline.** (PDF 94 kb)

## Abbreviations

DM: Type 2 diabetes mellitus
HbA1c: Glycated hemoglobin
HRV: Heart rate variability
BP: Blood pressure
HR: heart rate
LF: Low-frequency
HF: High-frequency
TP: Total power
